# The Role of Curiosity in Virtual Environments: A Conceptual Integration

**DOI:** 10.3390/bs14100899

**Published:** 2024-10-03

**Authors:** Rogelio Puente-Díaz

**Affiliations:** School of Business and Economics, Universidad Anáhuac, Huixquilucan 52786, Mexico; rogelio.puente@anahuac.mx

**Keywords:** curiosity, virtual environments, appetites, social goals, consumption goals

## Abstract

Brands need to have a digital strategy. Yet, it is difficult to grab consumers’ attention in virtual environments. We present the Curiosity in Virtual Environment (CVE) model, which integrates empirical and conceptual work on virtual environments, goal content, and curiosity. The model seeks to serve as a guiding framework and tool for research scholars and practitioners working in virtual environments who want to communicate with consumers. To elaborate and present the CVE, we first discuss the main characteristics of virtual environments and types of goal contents, followed by a brief introduction to the theoretical developments of curiosity. The model is then introduced, showing four quadrants in which practitioners should have an easier (more difficult) time grabbing consumers’ attention. We then conduct a selective review of experimental studies on curiosity and consumption, identifying three voids in the field. We finish the article by suggesting directions for future research and acknowledging the limitations of the CVE model.

## 1. Introduction

It is a marketing truism that brands should have digital strategies to communicate effectively with consumers and enhance their experience. Yet, the problem is that consumers do not naturally engage with brands’ efforts, as seen in the low clickthrough rates of most digital advertising and brand content on the Internet and social media [1]. In addition, virtual environments are saturated with brand information. This complex situation has been noted by business scholars, suggesting that the biggest challenge is to grab consumers’ attention [2]. Consequently, brands use different strategies to increase engagement with their brands and their advertising efforts in virtual environments. We posit that, explicitly or implicitly, brands try to elicit consumers’ interest and curiosity concerning their content. Brands want consumers to have interest and curiosity in their offerings, advertised promotions, and general knowledge. Hence, interest and curiosity are central targets of brands’ efforts. Brands want to grab consumers’ attention and then increase brand engagement. In addition, consumers hold different goal contents in their minds, which direct their attention. As the Internet has continued to grow and improve its development [3], understanding what motivates consumers and triggers their curiosity in virtual environments is crucial. In light of this, we propose a conceptual model that posits that three components need to be included to understand behavior in virtual environments: (1) the main characteristics of virtual environments, (2) the types of goal content held by consumers, and (3) the nature of the epistemic emotion of curiosity. All three components are needed to understand consumer behavior and help brands develop effective digital strategies that are capable of grabbing consumers’ attention. To reach the goal of elaborating our Curiosity in Virtual Environment (CVE) model, we briefly discuss the main characteristics of virtual environments, the importance of goal content in relation to consumer behavior, and the conceptual developments of curiosity. We finish the article by conducting a selective literature review of the strategies used to elicit curiosity in consumers and some directions for future research and limitations of our model.

Component 1: The main characteristics of virtual environments

Virtual environments have six important characteristics: (A) their social nature, (B) their high penetration and frequency of use, (C) their attractiveness for brands, (D) their most popular activities, (E) datafication, and (F) information overload. We organize this section around these six characteristics.

- Their social nature

From its early development, the Internet provided an environment for consumers to achieve different goals [4]. By far, social goals were more important, and they continue to be more important. Consumers could join interest groups, chat with friends and strangers, find lovers, and talk about their favorite movies and soap operas, among other social activities. The Internet quickly became a virtual social world, which represented an extension of consumers’ “real” social world.

Empirical studies and conceptual developments have shown the importance of social goals. One study examining reviews of Amazon suggested that even though consumers were engaging in economic behavior by evaluating the quality of books, it was clear that social goals in the form of community integration, bonding, establishing credibility, and seeking to stand out were prominent [5]. Another study showed that the top 500 videos viewed on YouTube only included three brands [6]. Hence, consumers show interest and curiosity about the content broadcast on YouTube, but their specific curiosity is not aimed at learning more about brands and their offers. The importance of social goals is further supported by research conducted on social platforms, such as Facebook, indicating that about 70% of consumers’ online activities involve checking the “timeline” and information about friends and others perceived as interesting [7]. Hence, searching for and looking at social information are consumers’ main activities on social media, entertainment platforms, and the Internet in general, with important implications for brands.

B. Their high penetration and frequency of use

The Internet, the use of smartphones, and social media apps have shown steady growth. The most recent data indicate that around 5.30 billion consumers have access to the Internet and around 5 billion use social media apps [3]. In addition, more than 5.61 billion consumers have a mobile phone [3]. In terms of the frequency of use, 96% of consumers use their smartphone within the first hour of being awake and spend several hours daily on it [3]. A recent survey found that searching for information and connecting with others are the two most important reasons to go online [3]. Interestingly, the same survey found that “killing time” is also a popular reason for going online [3]. Given the penetration of the Internet, social media apps, and smartphones, and how consumers interact with these devices, it is safe to conclude that virtual environments are popular places for consumers. Consequently, it is not surprising that brands find virtual environments attractive to reach and develop a relationship with consumers. Virtual environments are where brands need to be. Yet, it is important to point out that the high penetration and use also contribute to information overload (as discussed later on). It is not surprising then that grabbing the attention of consumers is becoming more challenging.

C. Their attractiveness to brands

Brands want to communicate with consumers. Hence, brands want to be where consumers are. Virtual environments represent great opportunities to reach consumers and establish relationships. Recent data show that spending on digital advertising has more than doubled in the past five years, and advertising spending on search engines and social media has the top two spots [3]. Yet, as several business scholars suggest, the main obstacle for brands is that consumers engage more with social information than brand content. In addition, there are thousands of brand messages trying to grab consumers’ limited attentional resources [8]. This has resulted in low levels of consumer engagement. Evidence shows that most digital advertising achieves low clickthrough rates [1]. Hence, virtual environments are attractive yet competitive. Brands need to take this into account when generating their digital strategies. In a way, brand content also contributes to the information overload in virtual environments. One important characteristic of the virtual environment relates to the most popular activities.

D. Their most popular activities

The virtual environment is too broad to cover all aspects. Consequently, we focus on social platforms or social media apps, online communities and blogs, and search engines, the three most popular online activities [1]. We posit that consumers show different goals and behaviors when engaging with social platforms, online communities, and search engines. Hence, the differences between these three should be considered.

### 1.1. Social Platforms

Social platforms are the main source of social life, especially for younger consumers [8]. When on social platforms, the main goal of consumers is to search for and obtain social information. Consumption information and information about brands are relatively less important. When on social platforms, consumers usually show high levels of interest and curiosity, which fuel their motivation to connect with others, consume and create content, and choose the type of content they want to pay attention to [9]. Given these characteristics, it is a challenging environment in which to attract consumers’ attention to brand content. Yet, brands need and want to have a presence on social platforms because this is where consumers spend a significant amount of their online time [3].

### 1.2. Online Communities and Blogs

Online communities and blogs could have social or brand-related information. Online communities could be organized around a social theme, a consumption theme, or a combination. Blogs could also be organized around social or consumption themes. One study suggests that the most popular blogs are related to consumption, where consumers discuss different topics from technology and fashion to celebrities and movies [10]. Consumers are likely to show naturally high levels of interest and curiosity in brand-related information, which could represent an opportunity for brands. Yet, we expect important individual differences as well. Specifically, online communities and blogs usually have two types of participants: active content generators and lurkers [11]. Active content generators and lurkers are likely to have important differences in their levels of interest and curiosity [11].

### 1.3. Search Engines

Compared to social platforms, online communities, and blogs, we could claim that the use of search engines is primarily energized by actively “looking for something” [1]. This “looking for something” is probably more related to consumption than social goals [1], which represents an opportunity for brands and their offerings. Yet, brands also face fierce competition because millions of brands have access to search engines and compete for the attention of consumers. Indeed, digital scholars posit that consumers often only pay attention to the first results provided by search algorithms, practically ignoring the rest of the search results shown [8].

Organic results are already a consequence of how interesting the content of a given website is. Interesting content often satisfies consumers’ interest and curiosity. Search engine algorithms, to some extent, consider how interesting a website is in the form of relevance and authority [1], two metrics used to determine the results after keyword searches. Paid or sponsored results come from auction bids [1]. Yet, brands still need to provide consumers with relevant information to satisfy their curiosity; otherwise, brands would be wasting their money by generating only minimal clickthrough rates.

E. Datafication

The last two characteristics, datafication and information overload, create a paradox. Brands have greatly benefited from datafication but, at the same time, the main challenge is the same: grabbing consumers’ attention. Both are discussed next.

Virtual environments are full of consumers’ information. Datafication is defined as converting into data online behaviors that were not measured, quantified, assessed, or understood previously [12]. The quantified behaviors in current virtual environments are likes, shares, sentiments, positive or negative comments, search volumes, and reputation indices, among others [13]. According to virtual communication scholars, datafication is a powerful tool used by brands to improve the precision of targeted advertising. We see at least two implications of datafication. First, datafication has greatly helped brands improve their advertising efforts. Yet, even if targeted advertising has improved its precision, brands still face the challenge of grabbing consumers’ attention because there are many brands competing for consumers’ attention, creating a paradox. Datafication is and will continue to be important. Yet, the challenge of grabbing consumers’ attention is and will persist in being relevant and one of the main obstacles faced by brands.

Second, datafication could provide rough, imperfect, yet relevant behavioral indicators of what is interesting to consumers to grab their attention. For example, one could argue that if consumers constantly click on, like, and share advertising messages, consumers might be interested in consumption-related information. They might be in a consumption mode, which represents valuable information for brands. Conversely, if consumers click on, like, and share messages coming from friends and interesting individuals, they might be more interested in social information. They might be in a social mode, which represents valuable information as well. In addition, if consumers’ amount of liking, sharing, and clicking in terms of social or consumption-related information is high, beyond a predetermined level, one could argue that their interest is higher than consumers showing a lower amount of liking, sharing, and clicking. Having these indicators will help brands know what consumers are currently interested in; yet, the challenge for brands remains almost the same: to grab consumers’ attention. The reason is simple: many brands will be interested in showing advertising to consumers, especially consumers in a consumption mode and showing high levels of interest as indicated by the information coming from the process of datafication. The main challenge of grabbing consumers’ attention is still relevant, which leads us to the last characteristic of virtual environments.

F. Information overload

The last defining characteristic of virtual environments is the amount of information, or information overload, available to consumers, including social and consumption-related information, which makes the challenge of grabbing consumers’ attention truly difficult [2,14]. Putting it simply, there is more information to attend to than available attentional resources [15]. Thus, the most valuable scarce resource is not money but consumers’ attention, with at least two important implications for brands [4]. First, it is harder to grab consumers’ attention due to information overload [2]. Thus, brands will need to spend more money to secure some type of attention, preferably quality attention. Indeed, the cost of grabbing the attention of consumers has grown significantly in the last decade, leading business scholars to coin the term “The Economy of Attention” to describe this challenge [2]. While the challenge of grabbing consumers’ attention is not only limited to the virtual environment, it is certainly a characteristic that needs to be considered when trying to generate interest in consumers. Second, the acknowledged challenge of grabbing consumers’ attention opens up the possibility of focusing on the content, or type of content, most likely to succeed. Content could be informative or entertaining [2], and efforts could be tailored depending on what consumers are looking for and their degree of attention. Thus, one way to grab consumers’ attention is by providing content that generates interest in consumers, with important implications for virtual environments and our conceptual model.

After discussing the six main characteristics of virtual environments and their implications, we are in a better position to discuss the concept of goals. The type of goal content is the second component of the Curiosity in Virtual Environments model.

Component 2: Consumer’s goals in virtual environments

Goals play an essential role in understanding consumer behavior in virtual environments [16] because goals energize and direct consumers’ motivation. The implications of this second component can be captured by two-subcomponents: (A) the influence of goals and (B) the type of goal content.

- The influence of goals

Goals are conceptualized as knowledge structures that influence important psychological processes such as attention, memory, information processing, attitudes, and judgments, among others [17]. These knowledge structures become activated, influencing what consumers pay attention to and remember, and their attitudes toward receiving new information. Given how competitive and saturated virtual environments are, knowing how goals influence attention, memory, and attitudes is important.

B. Goal content

Goal content also plays an important role in human motivation [18], reflecting what individuals want to do and achieve and the type of information they search for. Consumers can either have a social or consumption goal in mind. A social goal implies the desire to interact with others in virtual environments, to seek and obtain social information. A consumption goal implies the desire to interact with brands, or their content, to seek and obtain brand and consumption-related information. For the development of our conceptual model, we posit that goal content plays an important role in understanding why it is sometimes easier (more difficult) to grab consumers’ attention.

The main characteristics of the virtual environments and the goal content that consumers might seek are not enough to explain when and why brands should be able to attract consumers’ attention. We need to include a third component, examining the nature of curiosity, which is discussed next.

Component 3: The nature of curiosity

The third component involves three subcomponents: (A) the history and role of curiosity, (B) types of curiosity and their connections with appetites, and (C) implications for brands.

- The history and role of curiosity

The examination of curiosity has a long tradition dating back at least 70 years to the work of Berlyne [19], who proposed the distinction between perceptual and epistemic curiosity. Perceptual curiosity is related to sights, sounds, and images that draw interest. Epistemic curiosity involves being interested in learning new information. Brands could potentially elicit both types of curiosity.

Most experts on human development posit that individuals are born with the drive to explore their environment [20]. Hence, humans are curious by nature. Searching for and learning new knowledge is inherently pleasant. Curiosity is an epistemic emotion elicited by engaging in epistemic activities [21]. Epistemic activities include searching, inquiring, and learning new information [22]. Consumers in virtual environments usually search for social or consumption information. Thus, understanding the role of curiosity in virtual environments holds great potential.

B. Types of curiosity and their connections with appetites

Recent conceptual developments concerning curiosity include the distinction between interest-type curiosity and deprivation-type curiosity [23]. Interest-type curiosity is driven by the emotion of interest and is often connected with liking information (liking appetite system). Interest-type curiosity is energized by the anticipation of pleasure coming from searching and learning new information. Given that individuals are interested in learning new information, the urgency is not as intense as in the case of the second type of curiosity, the deprivation-type. The search for interesting information could be related to products and services, or consumption information, yet is more likely to be connected with social information, information about friends, relatives, and people of interest, just for the simple reason that in virtual environments, consumers tend to be more interested in social than in consumption information. This is an important point for understanding and explaining why consumers often do not want to engage with brand efforts and why consumers react negatively to interruptions by brands.

The second type of curiosity, deprivation-type curiosity, is driven by uncertainty and is often connected with wanting and needing information (wanting appetite system) [23]. Deprivation-type curiosity is energized by the need to search and learn needed information. It is conceptualized as a deficit, an uncomfortable realization of needing information. Hence, it is driven by negative emotions, as opposed to interest-type curiosity. In addition, as in interest-type curiosity, consumers could need information related to products and services or social information, information about friends and important others.

The distinction between interest- and deprivation-type curiosity, along with their respective appetite tendencies, liking, and wanting [23], could help us understand how consumers behave in digital environments and what type of brand efforts are likely to be ignored or attended to (see the work of [11] for some on the initial propositions of curiosity in digital environments). Similarly, this distinction could help us understand when the timing of brand efforts could be seen as useful or annoying, depending on whether consumers are driven by wanting and needing information or by liking new information and depending on whether consumers are searching for consumption or social information. This understanding could be useful, given that most brand efforts involve interrupting consumers [1].

C. Implications and challenges for brands

Generating curiosity is something that brands can utilize to their advantage in virtual environments. Yet, most experts on attention posit that we have limited resources [24], and salience stimuli are more likely to attract some of these limited resources [24]. Humans simply cannot pay attention to every brand effort nor can they be curious about each brand encountered on social platforms or the Internet. Consequently, brands have to battle for the attention of consumers, to battle for the interest and curiosity of consumers. This is why the conceptual developments of curiosity are relevant to understanding brands’ efforts in digital environments.

Connecting the main characteristics of virtual environments, component one, the types of goal content that consumers endorse, component two, and the types of curiosity with the respective appetite tendencies, component three, allows us to propose a conceptual model. In the next section, we present this conceptual model. We then selectively review the empirical literature and its implications for our model.

## 2. The Curiosity in Virtual Environments (CVE) Model

**Figure 1:**
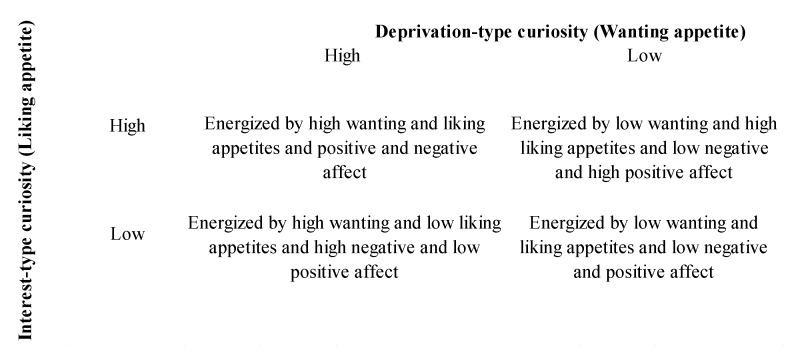
Conceptual model.

Our conceptual model includes the distinction between interest-type and deprivation-type curiosity, the distinction between the goal of obtaining social versus consumption- or brand-related information, types of appetites, liking versus wanting, and the assumption that curiosity varies along at least two levels, high or low, to simplify things and make the model workable. These multiple combinations, leading to four quadrants, allow us to propose different scenarios in which brands should have a more difficult or relatively easier time attracting the attention of consumers (see Figure 1).

1. High deprivation-type and interest-type curiosity for new information (high wanting and high liking)

In this first quadrant, consumers show high deprivation-type and high interest-type curiosity for new information, which are energized by high wanting and high liking. In this scenario, consumers perceive both a deficit in their information and a genuine interest in searching and obtaining new information. The search for information is driven by positive emotions such as interest and pleasure, or interest-type curiosity, and by negative emotions such as uncertainty and doubt, or deprivation-type curiosity. If consumers’ main goal is to obtain social information, they might not be receptive to brand efforts. Consumers’ high wanting and liking for social information does not put consumers in the mode of being receptive to consumption information. For brands to succeed in gaining the attention of consumers, they would have to present something highly original and useful to change consumers’ focus of attention. Specifically, brand efforts should be highly entertaining [2], useful (the deprivation part), or original and fun (the interest part) to attract consumers’ attention. Obtaining consumers’ attention is not the only obstacle that brands face. Consumers might react negatively to brand efforts if their goal is to obtain social information and brands interrupt or truncate consumers’ social goals (they want and like new social information). One empirical study suggested that when driven by high deprivation-type curiosity, consumers exhibit a thoughtful, careful approach to learning new information. In contrast, when driven by high interest-type curiosity, consumers exhibit a fun, carefree approach to learning new information [25]. Given that consumers show both high wanting and liking, these tendencies to process information differently might cancel each other out, being up to the originality and usefulness of brand efforts to elicit a careful or carefree approach to learning, assuming that brand efforts are able to attract consumers’ attention.

If the goal of consumers is to search for and obtain brand- or consumption-related information, consumers should be receptive to brand efforts. Brand efforts could be informative or entertaining [2]. Receptiveness should be even higher if brand efforts match the content of consumers’ goals. For example, if consumers search for information on women’s shoes on Instagram, consumers would be receptive to brand efforts showing discounts or information on a new, attractive brand of shoes. The challenge for brands is still to grab consumers’ attention because there are hundreds of brands providing information to consumers. Hence, the goal of attracting at least a minimum amount of attention from consumers, through clicking on an interesting ad, seems more reasonable than when consumers search for social information. Brands still need to compete for consumers’ limited attentional resources.

2. High deprivation-type and low interest-type curiosity (high wanting and low liking)

In this second quadrant, consumers show high deprivation-type and low interest-type curiosity, energized by high wanting and low liking appetites. In this scenario, consumers are mainly driven by the need for new information and negative affect in the form of uncertainty, doubt, or frustration. The perceived deficit and gap in knowledge is uncomfortable and consumers need to close this gap by searching for and obtaining information [26]. If the goal is to obtain social information, consumers would not be willing to switch their attention to brand efforts. Even highly original and useful brand information is likely to be ignored because the need for social information overcomes brand efforts. In addition, consumers might react negatively to brand efforts because consumers’ goals are social and driven by negative emotions. This is especially true if brands only follow an outbound strategy [1] in which consumers are constantly interrupted when seeking to obtain social information. This is probably the most challenging scenario for brands and it is more likely to be found on social platforms such as TikTok or Instagram. The low clickthrough rates of brand efforts observed on social media might be explained by high deprivation- and low interest-type curiosity when consumers want to search and obtain social information [7].

Conversely, if consumers need brand- or consumption-related information, consumers should be receptive to brand or consumption information to close their information gap. Yet, given that the wanting of information is high, consumers should pay closer attention to information that matches the content of their consumption goal (e.g., I am looking for Italian restaurants and TikTok is showing a relevant video of a new restaurant specializing in Italian-type pizza and pasta). Brand efforts should be informative [2] to help consumers close their knowledge gap. Consumers’ deprivation-type curiosity should lead consumers to aim at obtaining original and useful brand information. Conversely, if brand or consumption efforts do not match consumers’ goals, consumers might pay attention because they are in a “consumption mode” or if brand efforts are truly original and useful. Conversely, brand efforts might be ignored if consumers are not only driven by a high deprivation-type curiosity but also by time urgency (limited time available). The high deprivation-type curiosity, along with the wanting of information, opens up a new opportunity for brands in the form of inbound marketing strategies. Inbound marketing strategies generate useful content about brands and consumption [1], and consumers should not only be receptive to useful brand content but also spontaneously and voluntarily seek this type of information. Consumers should be appreciative of brand and consumption-related information, which helps alleviate their information gap.

3. Low deprivation-type and high interest-type curiosity (low wanting and high liking)

The third combination is one in which consumers show low deprivation-type and high interest-type curiosity energized by low wanting and high liking appetites for information. In this scenario, consumers are primarily driven by the interest, pleasure, and joy coming from searching for and learning new information. Consumers are likely to exhibit a carefree type of information processing. If consumers have a social goal in mind, their focus is on social and entertainment information. They might not be bothered by brand efforts, but they might not be highly receptive either, because the goal is social and their affect is positive. Brands need to make extremely appealing efforts to grab consumers’ attention. Brand content should be mainly entertaining [2]. Brands might also follow a social strategy in which brands help consumers obtain social information and play the role of the facilitator, with the potential of obtaining some indirect benefits [7].

If consumers have the goal of obtaining brand- or consumption-related information, consumers are in a receptive mode. Brand efforts should be informative [2]. They are more likely to be attracted to brand or consumption information consistent with consumers’ search content (I am looking for shoes, brand efforts about shoes are more relevant than efforts about jeans). Consumers would also be receptive to brand efforts not connected with their search content as long as they are perceived as original, useful, or informative to grab their attention. Both digital strategies, outbound and inbound, are likely to work because consumers have a high interest-type curiosity for new information.

4. Low deprivation-type and low interest-type curiosity (low wanting and low liking)

When consumers have low deprivation-type and low interest-type curiosity energized by low wanting and low liking appetites for information, consumers might be online out of boredom and seeking to just “kill” time. In this scenario, consumers are not driven by the need to close an information gap or by their interest in specific information. If consumers have a social goal in mind to cope with their boredom, they should not be receptive to brand efforts unless the efforts help consumers cope with their boredom. Brand content should be entertaining [2] to help consumers “kill some time”. If the goal is related to consumption, consumers might be receptive to brand efforts, but primarily if the efforts entertain consumers. However, their buying intentions might not be strong because their motivational drive is weak (low wanting and low liking). Research on virtual environments [3] suggests that “killing time” or “dealing with boredom” are common reasons to go online. Hence, marketers should consider the scenario of low deprivation- and interest-type curiosity for new information to attract consumers’ attention.

Our model seeks to integrate the conceptual developments on appetites and curiosity with consumers’ information goals, brand and consumption efforts, and the nature of virtual environments. We now review a selection of studies that have empirically tested the relationship between brand efforts and curiosity and how these findings map onto the model just described. The review is selective as opposed to exhaustive.

## 3. Empirical Studies

The purpose of reviewing the empirical literature was to examine how brand scholars have examined curiosity and its implications for virtual environments. Thus, we reviewed articles in which brand efforts had the goal of increasing consumers’ curiosity. These efforts usually focused on increasing engagement or buying intentions, among others, by eliciting curiosity. Given our interest in reviewing articles that wanted to increase consumers’ curiosity, we focused on only experimental studies, placing special emphasis on the mechanism through which curiosity was enhanced. We used the Web of Science search engine to find articles with the following keywords and their respective combinations: curiosity, marketing, consumer behavior, buying intentions, purchase intentions, and consumption. We limited our search to articles published since 2000 (when virtual environments started flourishing) and to experimental studies where researchers used different manipulations to increase curiosity. When reviewing the articles, we discussed how they fit our conceptual model.

### 3.1. Empirical Findings

In one of the pioneering studies assessing curiosity in advertising [27], researchers examined the influence of information gaps in advertising on curiosity. Participants were randomly assigned to three levels of knowledge gap: high, medium, and low. The results showed that participants exposed to advertising with a medium knowledge gap had higher curiosity than participants in the other two conditions. This enhanced curiosity led to more visits to the brands’ websites and to greater time spent on the websites. Participants were asked to simulate that they had a consumption goal in mind. It is unclear whether individuals with a social goal would react similarly to advertising with different levels of knowledge gap because advertisements would need first to grab consumers’ attention.

This second study is similar to the study just described but was conducted in the context of sports advertising [28]. The study was also based on the information gap conceptualization of situational curiosity. Participants were randomly assigned to one of three experimental conditions: high, medium, and low knowledge gap. The results showed that participants in the medium-level information gap condition showed greater curiosity than participants in the other two conditions. This enhanced curiosity led to greater intentions to watch the sport being advertised. Participants simulated having a consumption goal related to sports. It is unclear whether participants with a social goal would respond similarly to the three levels of knowledge manipulated in the advertising.

In one of the best-designed studies, researchers examined how moderate levels of mystery could increase curiosity, leading to greater purchase motivation and purchase intentions [29]. The argument was that moderate mystery should lead to greater curiosity than high or low mystery, based on the information gap conceptualization of deprivation-type curiosity. Moderate mystery was operationalized as providing consumers with some information but also withholding some information. The results robustly showed that moderate mystery led to higher curiosity, which then had a positive relationship with purchase motivation and intentions. Curiosity was treated as a mediator, explaining the process by which moderate mystery increased purchase motivation and intention. The study was conducted in a virtual environment in which participants simulated having a consumption goal. It is unclear whether this strategy would be effective if consumers had a social goal in mind. Yet, these findings showed that brands could “manipulate” the amount of information given to consumers to increase curiosity, with important implications for virtual environments.

This study examined how rumors and preannouncements can increase curiosity concerning new products [30]. Rumors usually come before preannouncements of new products. The basic premise was that rumors might instigate uncertainty, which might make consumers feel curious about the new product talked about in the rumor. In two experiments, the results showed that radical products led to greater curiosity, but only when the rumor about the product was ambiguous. By using radical versus incremental innovation in products, the researchers also included a degree of novelty in their experimental design. Curiosity then had a positive relationship with purchase intention. We could argue that participants had a consumption goal in mind, so we do not know how the rumor of a new product would grab the attention of consumers when they have a social goal in mind. Yet, we could conclude that if participants already have a consumption goal, rumors that are ambiguous, with an information gap, are able to generate greater curiosity for radical products, that is, products that truly show something novel.

When on social platforms, consumers are usually interested in engaging with information posted by friends and significant others. One study [31] tested the influence of group membership, with information coming from groups of friends or not, on buying intentions by increasing consumers’ curiosity. Consumers were shown an advertisement created by a brand on social media shared by friends or not. The results showed that posts on social media coming from groups of friends had a positive indirect influence on buying intentions by increasing curiosity. We could argue then that participants were simulated to be in a consumption mode, with a consumption goal on a social media app. It is not clear then if consumers with a social goal in mind would pay attention to the advertising even when it is shared by groups of friends. Yet, we posit that consumers want to socialize with their friends and brands focused on facilitating social interaction could benefit from these relationships in the form of virtual word of mouth and recommendations, among others [7].

After consumers decide to visit a website, we still observe a great deal of variability in the amount of engagement observed. In one study [32], researchers tested the role of virtual rooms in consumers’ engagement with websites. Curiosity was conceptualized as a mediator explaining how virtual rooms increase consumers’ engagement and buying intentions. In two experiments, the results showed that virtual rooms led to positive consequences in terms of buying intentions and exploration behavior. These positive outcomes were explained by increases in curiosity. For this particular study, participants were already engaged with a website for clothes and the researchers manipulated whether the website had a virtual room or not. Hence, consumers had a consumption goal activated and the virtual room enhanced the whole experience by increasing consumers’ curiosity.

This article is about incident curiosity [33]. The basic premise was that incidental curiosity would lead consumers to prefer unhealthy food. The article uses the deprivation type of curiosity approach to suggest that this aversive state of deprivation might lead consumers to engage in approach behavior, leading to choosing more rewarding food, which is unhealthy food. In three experiments manipulating incidental curiosity, the results showed that participants in the high curiosity condition reported higher levels of situational curiosity, which led to them choosing more unhealthy types of food. For this study, consumers had a consumption goal in mind by showing ads for cellphones to induce curiosity. It is unclear how this ad to induce curiosity would work if participants had a social goal in mind.

One study conducted in the domain of advertising tested whether an ad using augmented reality could lead to greater curiosity and attention, which would then lead to more positive brand attitudes [34]. Across one field experiment and two laboratory experiments, the results showed that participants exposed to the ad with augmented reality showed greater curiosity, paid greater attention to the ad, and developed more positive attitudes toward the ad than participants exposed to a regular ad (the control condition). For this study and its three experiments, participants already had a consumption goal in mind. Under the influence of this goal, consumers had more curiosity when exposed to the ad with augment reality, which translated to greater attention and more positive attitude toward the ad.

This specific study examined and tested different triggers of curiosity, information gap, ambiguity, and novelty on situational curiosity [35]. The researchers created ads with information gaps, ambiguities, novelties, and a control condition. Overall, across four experiments, the results showed that the information gap and ambiguity conditions generated greater curiosity than the other two conditions. Higher curiosity led to more positive brand evaluations due to increases in positive emotions and enhanced expectations. In these four experiments, participants were put in a consumption mode, so we could argue that they had a consumption goal. It is uncertain whether participants would notice the triggers used in advertising if they were browsing the Internet or social media with a social goal in mind.

In one study, curiosity was treated as a mediator to examine how congruency, that is, more or less congruent new products, influenced brand engagement by increasing curiosity [36]. Product congruency was conceptualized as the degree of congruency of a new product with previous products from the same brand. Participants were Twitter (now X) users. Participants were exposed to a Twitter message showing either a congruent or less congruent product for the brand Heinz. The results showed that less congruent products led to greater brand engagement by increasing consumers’ curiosity. The experimental protocol did not address whether consumers had a social or brand-related goal in mind. It did not address either whether participants were driven by deprivation or interest-type curiosity; yet, we could argue that a deficit in product knowledge caused by the less congruent product generated greater curiosity. The present study has implications for social media apps because, as mentioned earlier, it is difficult to attract consumers’ attention.

In another study [37], participants were asked to simulate that they were browsing on Instagram, searching for a dress (the participants were women). The study assumed that participants had a knowledge gap, deprivation-type curiosity, for novel products shown on Instagram and whether this knowledge gap led to higher curiosity and intentions was conditioned by the perceived socio-economic status of the women shown wearing the dresses. The results of two experiments showed that novelty had a positive direct influence on product curiosity and then intention, but only for moderate socio-economic status women shown wearing the dresses, as opposed to high socio-economic status women. In this study, participants were on social media with a consumption goal in mind. In this scenario, participants had more curiosity for novel products shown by women with a moderate socio-economic status.

The article is about framing a product, an idea, or something as reflecting change [38]. This framing could lead participants to feel curious about the product. Announcing change could trigger curiosity. The authors conducted seven experiments, a combination of field and laboratory experiments. In the first experiment, they manipulated whether the advertising included the word change and measured the clickthrough rates as a behavioral proxy of curiosity. The advertisement was launched on Facebook, increasing the external validity of the study. The clickthrough rates were low in general but significantly higher in the change condition as compared to the control condition. In the remaining laboratory experiments, the framing of change was compared to a control condition, and curiosity and interest in new information and buying intentions were assessed. The results showed that the framing of change led to greater curiosity, higher interest in new information, and higher buying intentions than the control condition. Consumers were likely to simulate having a consumption goal in mind.

The article focused on unconventional messages in packaging as a way to grab consumers’ attention [39]. The basic premise was that unconventional packaging messages activate an automatic thinking process, eliciting curiosity. They focused on impulse buying, conducting several experiments. In experiment two, participants were randomly assigned to an unconventional versus conventional message condition. The results in the unconventional condition showed that participants had higher levels of purchase intention, explained by increases in situational curiosity. The results of experiment three replicated the effect of unconventional messages on curiosity, but this effect was only significant when an emoji was present. The presence or absence of an emoji was a moderator. In this study, participants were asked to simulate that they were in a consumption mode with a consumption goal in mind. It is unclear whether the unconventional messages would be able to grab consumers’ attention and elicit curiosity when consumers have a social goal in mind.

The article argues that waiting for a product or a service can lead to higher curiosity with positive implications [40]. Curiosity was treated as a mediator. The premise was that a wait signals to consumers that something interesting is going on or something attractive is happening, enhancing their curiosity to find out. In experiment two, participants were randomly assigned to wait or not. Participants in the wait condition had higher buying intentions and those intentions were explained by higher curiosity. Experiment three also had two conditions, wait and not wait, and it was conducted in the context of museums. The results showed that participants in the wait condition had higher levels of willingness to visit the museum and this positive effect was explained by higher curiosity. In this well-conducted study, participants were in a consumption mode with a consumption goal in mind.

In an interesting study [41], participants were randomly assigned to a promotion versus prevention focus condition and exposed to vague or specific new product preannouncement (NPP), and the researchers assessed the effect of this interaction on curiosity and word of mouth. Participants were asked to imagine that they were browsing the Internet and encountered two types of new product announcements, vague versus specific, for a brand of smartphones. The results showed a positive influence of a vague NPP on curiosity and word-of-mouth intentions, but only for individuals who were induced to have a promotion-focused mindset. This study capitalized on the fact that vague preannouncements left out important details about the new product, generating an information gap that participants were curious to close. It is not entirely clear whether participants had a social or a consumption goal in mind because they were just asked to imagine that they were browsing the Internet. Yet, it is likely that this situation, just browsing the Internet, is a common situation that consumers face.

In another study worth noting for the combination of laboratory and field experiments [42], researchers tested the use of curiosity nudges to induce participants to choose should options versus want options in different domains. The conceptual development was based on the main characteristic of deprivation-type of curiosity, an information gap, and how satisfying this gap could only be achieved by choosing should options as opposed to want options. Across two laboratory experiments and two field experiments, the results showed a robust effect of curiosity nudges on the decision to choose should options rather than want options. This study was not conducted in a virtual environment, yet it does have implications. Marketers could use the findings of this study to design curiosity nudges and try to induce consumers to behave in a certain way. Marketers could capture consumers’ attention first by using curiosity nudges and then achieve the desired outcome.

In another study [43], participants were exposed to more creative or less creative packages, as previously validated in pilot studies, of music albums, and researchers examined the effect on curiosity, processing motivation, and then purchase intention. The results showed that participants exposed to more creative packaging showed higher levels of curiosity and then higher processing motivation, which translated to higher purchase motivation. While the experiment was not conducted in a virtual environment, it does have implications for virtual environments. Creative packaging could lead consumers to feel more curious and process information with higher motivation than less creative packaging. The exposure to more creative packaging could occur in virtual environments in which consumers could have either a consumption or a social goal.

After conducting searches, consumers have many links to engage with websites that might seem relevant. The degree of engagement with the chosen website can be enhanced by using virtual reality (VR). One empirical study [44] tested, in two experiments, the role of VR. The results showed that virtual reality immersive environments led to higher consumers having perceptual curiosity than non-immersive environments. This enhanced curiosity was positively related to greater consumer creativity. Both experiments were laboratory experiments in which participants were already in the act of shopping. We could argue that participants had a consumption goal in mind and either an interest-type or a deprivation-type of curiosity, given that it was not specified whether individuals needed to buy something or they were browsing shopping sites.

This article is on curiosity and advertising [45]. The main argument was that advertising that helps consumers close their information gap can make consumers less skeptical about the advertised products and the intentions of advertisers. The researchers manipulated exposing participants or not to advertising with information gaps and then assessed their curiosity and product evaluations. The results showed that advertising with information gaps led to greater curiosity than advertising with no information gap. When the brand helped alleviate this curiosity, participants felt less skeptical and evaluated the product more positively. The induced curiosity was specific to the product or incidental, and both had positive implications. Consumers were induced to have a consumption goal in some conditions and no goal in other conditions, extending the implications of these findings (see Table 1 for a summary of the experiments reviewed).

### 3.2. Main Results

After reviewing 19 experiments in which the goal was to increase curiosity, we can draw the following conclusions. (1) Most studies were successful at increasing curiosity. Practitioners can achieve the goal of increasing consumers’ curiosity in virtual environments, and our review showed what types of experimental manipulations were likely to succeed. (2) In almost all the studies, researchers had participants simulate that they had a consumption goal in which the stimuli shown were already assumed to be relevant to consumers. These studies did not have stimuli competing for consumers’ attention. This is an important omission because virtual environments are characterized by information overload [2,14]. (3) The manipulation of whether participants had a high or low level of either deprivation- or epistemic-type curiosity was absent. In other words, not a single experiment manipulated the urgency or the need for the consumption type of information. (4) Most studies, after assessing curiosity, focused on purchase intentions or brand evaluations, without considering additional behavior outcomes related to actual consumer behavior. The lack of emphasis on examining how consumers with a social goal in mind will respond to different stimuli designed to increase curiosity, the lack of manipulating how urgent or needed the information is for consumers, and limited emphasis on behavioral outcomes represent important research limitations and opportunities for the future.

## 4. Future Directions

Combining our conceptual model with the empirical findings just reviewed, we see three directions for future research: conducting experiments in which social goals are included, manipulating how urgent or needed the information is for consumers, and examining additional outcomes beyond intentions and brand evaluations.

A. Experiments with social goals

As mentioned before, in almost all the experiments, participants were asked to simulate that they had a consumption goal, that they wanted to buy or try on a piece of clothing. Future studies could ask participants to simulate that they have a social goal in mind. Researchers could ask participants to simulate that they have a social goal of seeking information on what their friends did this past weekend and expose participants to advertising with knowledge gaps or ambiguous information and examine whether an information gap or ambiguous information is enough to attract consumers’ attention. Based on conceptual developments and empirical studies, we predict low levels of engagement when consumers have a social goal in mind. Yet, it is still relevant to understand if these low levels of engagement differ as a function of the types of advertising shown, information gap versus complete information. These types of experiments will also have the advantage of creating more realistic scenarios, with greater ecological validity.

B. Manipulating urgency or how needed the information is

Consumers spend several hours daily in virtual environments. Future studies could manipulate how urgent or needed information is for consumers and then expose participants to different stimuli trying to elicit curiosity. Based on our conceptual model, we predict that when consumers have a high deprivation type of curiosity, that is, truly needing social information, it will be difficult to elicit curiosity with consumption information. If consumers have a high deprivation type of curiosity for consumption information, it will be easier to generate curiosity and more likely to generate curiosity when the information searched by consumers matches the information shown. Conducting these types of experiments will generate relevant information for researchers and practitioners interested in understanding the role of curiosity in virtual environments.

C. Assessing additional outcomes beyond intentions.

Thus far, the empirical literature has focused almost entirely on intentions and brand evaluations or other self-report outcomes. The literature has been useful in assessing how induced curiosity usually has positive relationships with buying intentions and brand evaluations, among others. Yet, beyond these self-report outcomes, we know almost nothing about the actual buying behavior of consumers. Researchers could assess other relevant outcomes, such as time searching for information, browsing the web, and time spent on the websites of the advertised brands, among others. These additional outcomes could be conceptualized as epistemic activities seeking to “know more” about the advertised brand, driven primarily by curiosity. These additional outcomes could provide researchers and practitioners with relevant information to examine curiosity and its implications for consumer behavior.

## 5. Conclusions

Nowadays, the biggest challenge for brands is to grab consumers’ attention in virtual environments due to six relevant characteristics: (A) their social nature, (B) their high penetration and frequency of use, (C) their attractiveness for brands, (D) their most popular activities, (E) datafication, and (F) information overload. We also proposed that consumers have two main goals: social and consumption-related. The core of our model is the proposition that curiosity, an epistemic emotion, plays an important role in virtual environments. We developed a model that included the latest developments concerning types of curiosity, virtual environments, and types of goals that consumers strive for. After reviewing the empirical literature, we provided some directions for future research. We posit that the exploration, examination, and conceptualization of the role of curiosity in virtual environments hold great potential for business scholars, practitioners, and researchers. We hope our colleagues share our excitement about the role of curiosity in virtual environments.

## Figures and Tables

**Table 1:** Summary of experiments reviewed.

Article	Type of Goal	Conceptualization	Main Finding
Menon & Soman [27]	Assumed to have a consumption goal	Knowledge gap	Works best to have moderate knowlege gap to elicit curiosity
Park et al. [28]	Assumed to have a consumption goal	Knowledge gap	Works best to have moderate knowlege gap to elicit curiosity
Hill et al. [29]	Assumed to have a consumption goal	Knowledge gap	Moderate mistery elicits higher curiosity
Sääksjärvi et al. [30]	Assumed to have a consumption goal	Knowlege gap	Ambiguous rumors lead to greater curiosity
Thomas & Vinuales [31]	Assumed to have a consumption goal while in social media	Knowledge gap	Posts coming from friends elicit greater curiosity
Beck, M., & Cri, D. [32]	Assumed to have a consumption goal	Perceptual curiosity	Virtual fitting rooms elicited greater curiosity
Wang [33]	Assumed to have a consumption goal	Knowlege gap, incidental curiosity	Incidental curiosity leads to greater choosing of unhealthy products
Yang et al. [34]	Assumed to have a consumption goal	Perceptual curiosity	Augmented reality in advertising increases curiosity
Daume & Hüttl-Maack [35]	Assumes to have a consumption goal, Paper and pencil	Knowledge gap	Advertising with knowledge gap and ambiguity lead to greater curiosity
Gerrath & Biraglia [36]	Assumed to have a consumption goal in social media (X)	Knowledge gap	Less congruent brands led to greater curiosity
Shin & Lee [37]	Assumed to have a consumption goal	Knowledge gap	Product novelty leads to greater curiosity
Kupor et al. [38]	Assumed to have a social goal while in social media	Knowledge gap	The reference to change leads to greater curiosity
Das et al. [39]	Assumed to have a consumption goal	Knowledge gap	Unconventional messages in packagging leads to greater curiosity
Wang et al. [40]	Assumed to have a consumption goal	Knowledge gap	Creating a wait leads to greater curiosity
Wang et al. [41]	Assumed to have a consumption goal	Knowledge gap	Vague new product preannouncements generated greater curiosity
Polman et al. [42]	Assumed to have a consumption goal and also no goal assumed	Knowledge gap	Nudges previously tested as curiosity eliciting lead to more positive consumer behavior
Shukla et al. [43]	Assumed to have a consumption goal	Perceptual curiosity	More creative product packages lead to greater curiosity
Kim & Choo [44]	Assumed to have a consumption goal	Perceptual curiosity	Virtual reality elicits more perceptual curiosity
Hüttl-Maack et al. [45]	Assumed to have a social and consumtion goal	Knowledge gap and its resolution	Creating a knowledge gap leads to greater curiosity. Solving the curiosity leads to positive brand evaluations and less skepticism

## Data Availability

This study did not collect any data.
